# Microglial-associated responses to comorbid amyloid pathology and hyperhomocysteinemia in an aged knock-in mouse model of Alzheimer’s disease

**DOI:** 10.1186/s12974-020-01938-7

**Published:** 2020-09-17

**Authors:** David J. Braun, Edgardo Dimayuga, Josh M. Morganti, Linda J. Van Eldik

**Affiliations:** 1 Sanders-Brown Center on Aging, University of Kentucky, 101 Sanders-Brown Bldg., 800 S. Limestone Street, Lexington, KY 40536 USA; 2 Department of Neuroscience, University of Kentucky, Lexington, KY USA; 3 Spinal Cord and Brain Injury Research Center, University of Kentucky, Lexington, KY USA

**Keywords:** Alzheimer’s disease, Amyloid, Homocysteine, Hyperhomocysteinemia, Microglia, Neuroinflammation

## Abstract

**Background:**

Elevated blood homocysteine levels, termed hyperhomocysteinemia (HHcy), is a prevalent risk factor for Alzheimer’s disease (AD) in elderly populations. While dietary supplementation of B-vitamins is a generally effective method to lower homocysteine levels, there is little if any benefit to cognition. In the context of amyloid pathology, dietary-induced HHcy is known to enhance amyloid deposition and certain inflammatory responses. Little is known, however, about whether there is a more specific effect on microglia resulting from combined amyloid and HHcy pathologies.

**Methods:**

The present study used a knock-in mouse model of amyloidosis, aged to 12 months, given 8 weeks of B-vitamin deficiency-induced HHcy to better understand how microglia are affected in this comorbidity context.

**Results:**

We found that HHcy-inducing diet increased amyloid plaque burden, altered the neuroinflammatory milieu, and upregulated the expression of multiple damage-associated and “homeostatic” microglial genes.

**Conclusions:**

Taken together, these data indicate complex effects of comorbid pathologies on microglial function that are not driven solely by increased amyloid burden. Given the highly dynamic nature of microglia, their central role in AD pathology, and the frequent occurrence of various comorbidities in AD patients, it is increasingly important to understand how microglia respond to mixed pathological processes.

## Background

Homocysteine (Hcy) is an intermediary in essential cellular pathways, and its metabolism depends on several vitamin cofactors, primarily B_6_, B_9_ (folic acid), and B_12_. Deficiencies in one or more of these are therefore a common cause of elevated blood homocysteine, termed hyperhomocysteinemia (HHcy) [1]. Evidence indicates that both elevated homocysteine itself, and the broader metabolic dysfunctions resulting from B-vitamin deficiency, are relevant to its status as a vascular risk factor and risk factor for Alzheimer’s disease (AD) [2–4]. The normal human range for Hcy in plasma is generally considered to be less than 15 μM, with increasing levels categorized as moderate (15–30 μM), intermediate (30–100 μM), or severe (> 100 μM) HHcy. Although the prevalence of HHcy in the general population is estimated to be around 5%, it is significantly higher in the elderly [5], a population in which B-vitamin status is a major contributor to HHcy [6]. Interestingly, elderly men have been found to have elevated plasma homocysteine compared to women [7], while AD is more common in women [8], highlighting that HHcy is only one in a complex array of factors influencing any given individual’s risk of dementia. Nonetheless, the population attributable risk of dementia from raised Hcy is estimated to be between 4 and 31%; in other words, preventing HHcy could prevent somewhere between 1 in 25 to 1 in 3 cases of AD [9]. In the USA alone, even the conservative estimate would translate to hundreds of thousands of people. It follows, then, that reduction of HHcy in the population is an attractive goal. In addition to lifestyle modifications (e.g*.,* quitting smoking, exercise), B-vitamin supplementation is a fairly straightforward intervention that reduces circulating Hcy levels and thus the prevalence of HHcy [10]. Encouragingly, since the USS began supplementing grain with folic acid in the late 1990’s, the prevalence of HHcy has roughly halved [11], and this could potentially be a contributor to the recently reported reductions in the rate of increase in AD incidence [12]. Nonetheless, correcting the acute metabolic dysfunctions underlying elevated Hcy appears to be a necessary but insufficient step to abrogate the full contribution of this risk factor to AD-associated cognitive decline [9], and adjunct treatments will likely be important moving forward.

HHcy-related neural dysfunction is a multifactorial pathological process, mediated by complex and interacting pathways related to oxidative damage, inflammation, hypomethylation, and others [13]. In this regard, it shares commonalities with AD, and there may be important points of convergence amenable to therapeutic targeting. Recent work has highlighted the role of microglia as a nexus in the development and progression of AD pathology (for review see [14]), with particular focus on a shift of microglia from a more “homeostatic” phenotype to a damage-associated or neurodegenerative (DAM) phenotype [15, 16]. The microglial responses to pathological Hcy elevation are less well-characterized, but there is evidence that homocysteine directly increases activation of microglial-type cells in vitro [17, 18], and both direct Hcy supplementation [19] and vitamin deficiency-induced HHcy have similar impacts in vivo [20–22]. Microglial dysregulation could therefore represent an important convergence point in the context of comorbid HHcy and amyloid pathology. There are several major possibilities here that are relevant from a treatment standpoint and not mutually exclusive: the comorbidity context accentuates dysfunctional microglial responses to amyloid, it induces more of the microglia to assume a dysfunctional DAM state, or it causes some emergent alteration unique to these comorbid pathologies. The goal of the present study is to better understand these possibilities by using a knock-in (KI) mouse model of amyloidosis with mutant presenilin 1 and amyloid precursor protein transgenes under control of endogenous promoters to avoid potential overexpression artifacts [23]. Male and female mice aged 12 months were placed on B-vitamin deficient diet for 8 weeks, with the effect on various neuroinflammatory and microglial parameters characterized at the conclusion of dietary administration, when mice were about 14 months old. We found that elevation of homocysteine level in the context of amyloid pathology enhances parenchymal plaque deposition, subtly alters the neuroinflammatory milieu, and influences a number of genes important to microglial functioning. Interestingly, these changes are consistent with both an increase in DAM microglia as well as some emergent effects on microglia-relevant pathways. This study lays the groundwork for follow-up experiments making use of cell-specific isolation and next generation sequencing technologies.

## Methods

### Animals and experimental design

All mice were housed 1–5 per cage (503.22 usable cm^2^) in a room at 23 °C ± 2 °C, under a 14/10-h light/dark cycle beginning at 6:00 AM, with *ad libitum* access to water and chow. The APP^NLh/NLh^ × PS1^P264L/P264L^ double knock-in (KI) mouse model expresses humanized amyloid precursor protein with the Swedish mutation (K670N/M671L), along with a P264L point mutation in the mouse presenilin 1 gene [24]. The homozygous KI mice were maintained on a combined CD-1/129 background, with wildtype (WT) controls derived separately from matings of heterozygous KI animals. The experiment was carried out in a 2 × 2 diet by genotype design, where half the mice of each genotype were randomized to receive 8 weeks of control (Envigo, #TD.01636) or HHcy diet (Envigo, #TD.97345), beginning between 52 and 54 weeks of age (*M* = 53.4 weeks, SD = 0.36). The HHcy diet was deficient in vitamins B6, B9, and B12 and supplemented with excess methionine, while the control diet was nutritionally matched with normal methionine and B-vitamin levels [25]. Humane-endpoint criteria were 20% weight loss across the study or 10% weight loss within two weeks, along with detrimental changes in body condition (e.g., lethargy, hunched posture, dehydration, dermatitis). None of the mice reached humane-endpoint criteria; however, three mice died during the study and were excluded from all analyses: one KI female receiving HHcy diet, one WT male receiving control diet, and one WT male receiving HHcy diet. Importantly, mice were randomized irrespective of sex, resulting in an imbalance in the final sex ratios. The final numbers of mice for each group were 8 WT on control diet (5 female and 3 male), 10 WT on HHcy diet (5 female and 5 male), 11 KI on control diet (4 female and 7 male), and 10 KI on HHcy diet (8 female and 2 male). As the experiment was not powered to detect sex differences, it should be noted that the imbalance may have increased experimental variability and thus reduced statistical power, or otherwise influenced the data. The experiment was performed in compliance with the Institutional Animal Care and Use Committee of the University of Kentucky.

### Tissue collection and blood analyses

Mice were deeply anesthetized with 5% isoflurane, and arterial blood collected from the left ventricle and placed into EDTA-plasma tubes (Greiner Bio-One, #454428) for separation of plasma by centrifugation (2000×*g* for 20 min at room temperature) followed by storage at − 80 °C. As an interim analysis to verify the expected dietary effects, a subset of 7 WT mice (4 on HHcy and 3 on control diet) had whole blood taken for hematologic analysis using i-STAT CG8+ cartridges (Abbott Laboratories) according to the manufacturer’s instructions. Additionally, at the conclusion of the full study, plasma samples from a randomly chosen subset of 5 mice per group (20 total) were diluted 1:5 in ARCHITECT Multi-Assay Manual Diluent (Abbott Laboratories, Chicago, IL, USA) and delivered to the University of Kentucky Clinical Laboratory for Hcy measurement on an ARCHITECT i2000SR analyzer (Abbott Laboratories). Samples were diluted to reach the volume requirements for the assay (500 μL). The lower limit of detection is 1 μM, and diluted samples reading less than 1 do not give an output value. Samples with an output value > 1 μM were multiplied by the dilution factor to determine the total homocysteine concentration in the undiluted plasma. None of the diluted samples from mice on control diet gave a reading of greater than 1 μM.

All mice subsequently underwent transcardial perfusion with 50 ml ice-cold phosphate-buffered saline (PBS) at a flow rate of 10 ml/min before decapitation and brain removal and dissection. The right hemisphere was post-fixed in 4% paraformaldehyde for 24 h at 4 °C and cryo-protected in 30% sucrose for at least 48 h at 4 °C. Samples were subsequently cut into 30 μm sections with a sliding microtome and stored in cryoprotectant solution at − 20 °C prior to immunofluorescent staining. The hippocampus and overlying cortex were dissected from the left hemisphere, flash frozen in liquid nitrogen, and stored at − 80 °C until processing for biochemical endpoints described below. The cortex was divided in two: a more caudal piece directly overlying the hippocampus was used for protein analysis. An adjacent rostral piece was used for mRNA expression analysis. A portion of dorsal hippocampus was used for protein analysis. See supplement 1 for an illustration of brain regions dissected and outlined for analysis.

### Immunofluorescence

Staining was performed on free-floating sections spaced approximately 300 apart through the dorsal hippocampus and overlying cortex, for a total of 6–8 sections per animal. Blocking was performed with 10% normal goat serum (Lampire Biological Laboratories, #7332500) and 0.2% Triton X-100 in PBS. All antibodies were diluted in PBS with 3% normal goat serum and 0.2% Triton X-100. Sections were incubated overnight at 4 °C with rabbit anti-P2ry12 (1:500, Anaspec #AS-55043A) and mouse anti-Aβ 6E10 conjugated to Alexa 647 (1:200, BioLegend #803021). Samples were subsequently incubated at room temperature for 2 h in 1:500 secondary antibody solution with Alexa 488 goat anti-rabbit (Invitrogen, #A-11034). Sections were mounted and treated for 5 min with 1x TrueBlack (VWR, #10119-144) in 70% ethanol to reduce autofluorescence before drying and coverslipping in Vectashield mounting medium with DAPI (Vector Laboratories, #H-1200). Entire slides were imaged on a Zeiss Axio Scan Z1 digital slide scanner at × 20 magnification.

### Image analysis

The dorsal hippocampus and overlying cortex were manually outlined in the HALO analysis suite (Indica Labs, version 2.3.2089.34) by an investigator blinded to experimental groups. The algorithm minimum intensity settings for all analyses were manually thresholded based upon negative control (no primary antibody). For cortical and hippocampal analyses of P2ry12 staining, and P2ry12 and 6E10 co-localization, the positive pixel algorithm (Area Quantification FL v1.2) was applied to the traced region across all sections per animal to give a single value of total percent area stained per region per mouse. For cortical and hippocampal analyses of 6E10 staining, the object counter algorithm (Object Colocalization FL v1.0) was applied to the traced region across all sections to give a single average count per square millimeter of tissue per region. Minimum object size was set to 10 μm^2^. Plaque size distributions were analyzed in two ways. First, plaques were binned into small (< 300 μm^2^), medium (301 to 900 μm^2^) and large (901+ μm^2^) size ranges. Arbitrary cutoff points were based on a previous study of this model [26], and the average number of plaques/mm^2^ within each bin were determined per animal and compared between diet groups. Second, cumulative size distribution curves were generated for each diet by treating each plaque as an individual data point within each dietary condition, and testing the curves for difference with the Kolmogorov-Smirnov test [27]. For the spatial analysis of microglia around amyloid plaques, 1–4 individual plaques were manually circled per section within the outlined cortical region, totaling 15 plaques per animal or 120 per diet condition. This number was based upon the plaque sampling rate used in the previous reference [24] of about 100 per condition. The circle annotation tool was used to manually circle the entirety of the plaque, and 3 outward concentric partitions added with a fixed width of 30 μm. The positive pixel algorithm was applied within each ring (inner, middle, and outer). To be selected, plaques had to be far enough away from tissue edges or other plaques to enable non-overlapping ROIs completely contained within the tissue. The semi-randomly selected plaques had a significantly larger median size (215.6 μm^2^) than the full distribution (51.5 μm^2^), likely because the smallest plaques tended to be more clustered and therefore not amenable to this analysis. Nonetheless, the median size of the plaques analyzed did not differ between the control (293.7 μm^2^) or HHcy (219.8 μm^2^) dietary groups (two-tailed Mann-Whitney *U*, *p* = 0.235). The cumulative size distributions of the circled plaques were also not significantly different between the two groups (Kolmogorov-Smirnov, *D* = 0.099, *p* = 0.482).

### MesoScale Discovery (MSD) multiplex ELISA

Pieces of the dissected hippocampus and cortex from the left hemisphere were used that approximate the regions outlined for the immunofluorescence analyses in the right hemisphere. Tissues from each mouse were homogenized using an Omni Bead Ruptor 24 (Omni International) at a 1:20 weight to volume ratio in lysis buffer: PBS with 1 mM PMSF, 0.5 mM EDTA and 0.2X Halt Protease Inhibitor Cocktail (Thermo Scientific, #87786). Homogenates were centrifuged at 12,000×*g* for 20 min at 4 °C. Supernatants were collected for cytokine or Aβ_1–40_ (Aβ40)/Aβ_1–42_ (Aβ42) measurement using MSD custom mouse V-Plex ELISA kits and a human 6E10 Aβ kit (K15200E), respectively. IL-1β, IL-6, TNFα, and CXCL1 were run multiplexed as part of the Proinflammatory Panel 1 Mouse Kit (K15048). CCL3 and IL-33 were separately run multiplexed as part of the Cytokine Panel 1 Mouse Kit (K15245). All samples were run undiluted. For the cytokines, samples were incubated overnight at 4 °C. Samples were incubated for 2 h at room temperature for the Aβ kit. Cytokine levels and Aβ peptide levels were normalized to the total milligrams (mg) of protein loaded in the sample as determined by BCA Protein Assay (ThermoFisher #23225).

### Quantitative reverse-transcriptase polymerase chain reaction (qRT-PCR)

RNA was isolated from the cortical tissue piece just rostral to that used for protein extraction with the RNeasy Plus Mini Kit (Qiagen, #74136) according to the manufacturer’s instructions. Tissue was weighed and homogenized in an appropriate volume of buffer, and genomic DNA removed with the gDNA eliminator column. The samples were mixed with 70% ethanol, run through the RNeasy column, washed, and eluted in RNase-free water. Quantity and quality of RNA and 260/280 absorbance ratios were assessed using a NanoDrop spectrophotometer (ThermoFisher Scientific). Reverse transcription was performed with the High Capacity cDNA Reverse Transcription kit (Applied Biosystems, #4368814) according to the manufacturer’s protocol. Real-time PCR was performed on a ViiA 7 Real-Time PCR System (Applied Biosystems) using individual TaqMan® probes for *P2ry12*, *Clec7a*, and *Itgax* and custom TaqMan®Array Cards (ThermoFisher Scientific, #4342253) with TaqMan Fast Advanced Master Mix (ThermoFisher Scientific, #4444557). See Table 1 for a complete list of genes and probes included on the array. Established microglial markers affected by amyloid pathology were chosen based on recent studies [15, 16]. Relative gene expression was calculated using the 2^−∆∆CT^ method and log2 normalized. HPRT and 18S were used as housekeeping genes for the individual probes and the custom array, respectively.

**Table 1:** Gene names and TaqMan probes analyzed in this study. Genes in bold were run with individual probes: the rest were run together on TaqMan custom low-density array cards

Gene	TaqMan probe
Apoe	Mm01307193_g1
B2m	Mm00437762_m1
C1qa	Mm00432142_m1
C1qb	Mm01179619_m1
C1qc	Mm00776126_m1
Ccl2	Mm00441242_m1
Ccrl2	Mm00516914_g1
Cd74	Mm00658576_m1
Chil3	Mm00657889_mH
Csf1	Mm00432686_m1
Csf1r	Mm01266652_m1
Cst3	Mm00438347_m1
Cst7	Mm00438351_m1
Ctsz	Mm00517697_m1
Ctsb	Mm01310506_m1
Ctsd	Mm00515586_m1
Ctsl	Mm00515597_m1
Ctss	Mm01255859_m1
Axl	Mm00437221_m1
Cx3cr1	Mm02620111_s1
Cyba	Mm00514478_m1
Eef1a1	Mm01973893_g1
Egr1	Mm00656724_m1
P2ry13	Mm01951265_s1
F11r	Mm00554113_m1
Fcrls	Mm00472833_m1
Ftl1	Mm03030144_g1
Fth1	Mm00850707_g1
Golm1	Mm00550918_m1
Gpr34	Mm02620221_s1
Hexb	Mm01282432_m1
Hif1a	Mm00468869_m1
Kctd12	Mm00624816_s1
Lag3	Mm00493071_m1
Lyz2	Mm01612741_m1
Mafb	Mm00627481_s1
Olfml3	Mm00513567_m1
Pde3b	Mm00691635_m1
Plxdc2	Mm00470653_m1
Sall1	Mm00491266_m1
Slc11a1	Mm00443045_m1
Slco2b1	Mm00614448_m1
Spp1	Mm00436767_m1
Tgfbr1	Mm00436964_m1
Tpt1	Mm03009502_g1
Tyrobp	Mm00449152_m1
Mertk	Mm00434920_m1
18S	Hs99999901_s1
**Clec7a**	**Mm01183349_m1**
**P2ry12**	**Mm00446026_m1**
**Itgax**	**Mm00498701_m1**
**Hprt**	**Mm00446968_m1**

### Statistical analysis, figure generation, and reporting

Analyses and figure generation were performed with JMP Pro 14 (SAS) and Prism 8.3.0 (GraphPad). Two-way analysis of variance (ANOVA) with Sidak’s or Dunnett’s post hoc testing, student’s *t* tests, or non-parametric Mann-Whitney *U* tests as indicated in the figure legends or text. For plaque size analysis, Kolmogorov-Smirnov tests were used to determine whether the cumulative plaque size distributions differed between dietary groups. For the gene expression data, three group comparisons were made: WT HHcy versus WT control, KI control vs WT control, and KI HHcy versus KI control. To generate the heatmap, group scores were averaged and transformed into *z*-scores using JMP; then, the variable clustering script was run to group genes by similarity of expression pattern prior to visualization in Prism. Where reported in the text, group means are followed by the standard deviation (SD).

## Results

### Eight weeks of B-vitamin-deficient and methionine-supplemented diet induces HHcy and increases plaque deposition in the KI mice

**Fig. 1:**
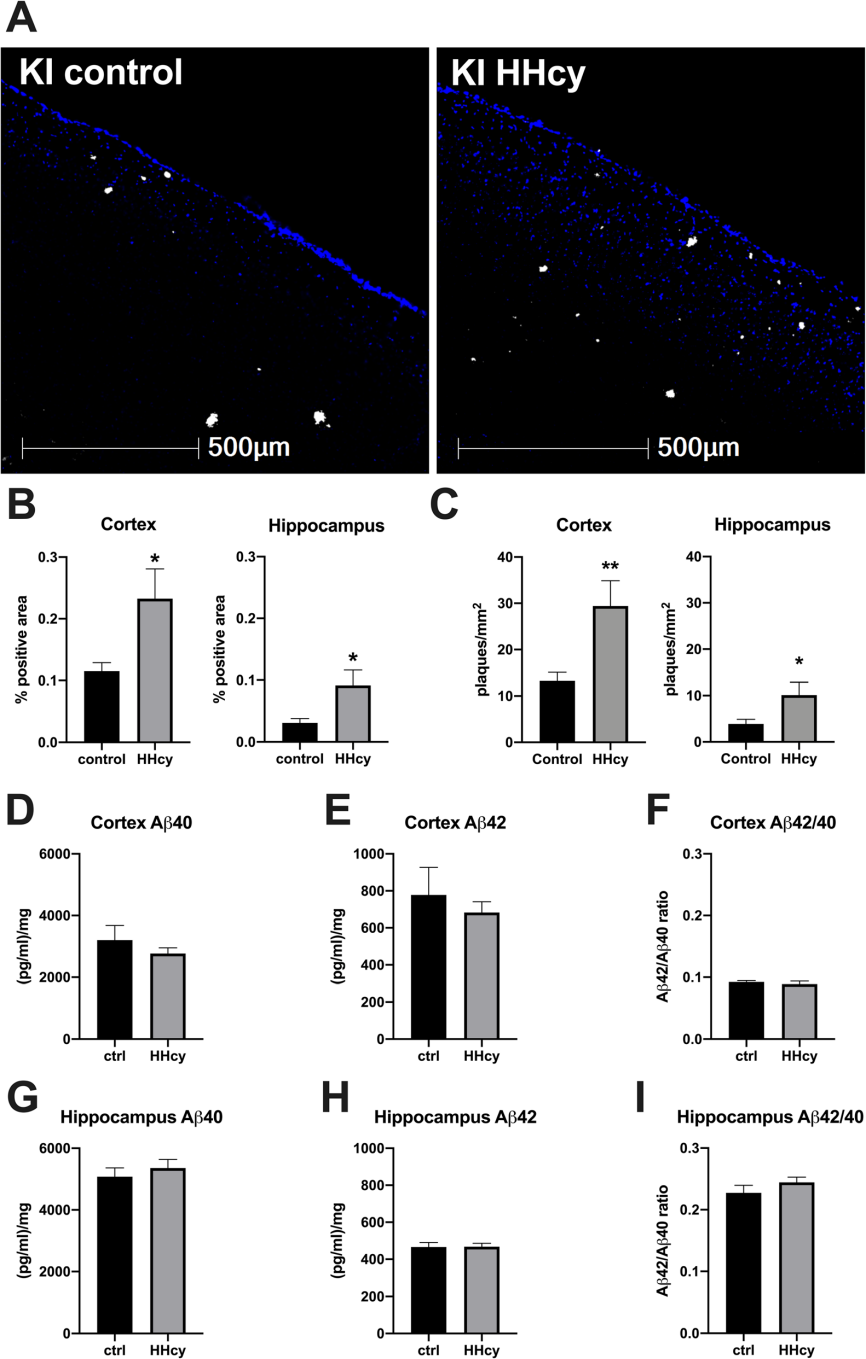
Dietary-induced HHcy is associated with increased cortical and hippocampal plaque burden in KI mice. **a** Representative images of dorsal cortex from KI mice on control diet (KI control) or HHcy diet (KI HHcy). Nuclei were stained with DAPI and are shown in blue; amyloid beta plaques were stained with 6E10 antibody and are shown in white. **b** Total 6E10 % positive area within cortical and hippocampal ROIs is quantified, showing a statistically significant increase in cortex and hippocampus. The increase in % area positivity for 6E10 corresponded with an increase in the number of plaques per square millimeter in the cortex and hippocampus (**c**). Levels of soluble Aβ40, Aβ42, and the Aβ42/40 ratios were unchanged in both the hippocampus and cortex (**d**–**i**). *p < .05, **p < .01, non-parametric Mann-Whitney *U* tests

The average plasma homocysteine levels after HHcy diet were similar between genotypes, with WT mice achieving an average concentration of 19.0 μM (SD = 6.8 μM) versus 14.7 μM (SD = 2.8 μM) in the KI animals (for full dietary validation data see supplement 2). The plasma samples from all mice on control diet were below the limit of detection for the test after dilution—meaning the undiluted samples were less than 5 μM—consistent with what has been reported in the literature by ourselves and others [25, 28]. Additionally, we found that in the KI mice HHcy was associated with greater amyloid plaque burden in the cortex and hippocampus, as measured by 6E10 percent area positivity, and corresponding with an increase in the total number of plaques (Fig. 1a–c). In the cortex, average plaque number increased from 13.3 per mm^2^ (SD = 6.1) in KI mice on control diet to 29.4 mm^2^ (SD = 17.3) in those on HHcy diet. Hippocampal plaques increased from 3.9 (SD = 3.3) to 10.1 (SD = 8.7) per mm^2^ in mice on control or HHcy diet, respectively. No changes were detected in hippocampal or cortical levels of Aβ40, Aβ42, nor the Aβ42/40 ratio as measured by ELISA (Fig. 1d–i). Notably, no evidence of cerebral amyloid angiopathy (CAA) was found.

**Fig. 2:**
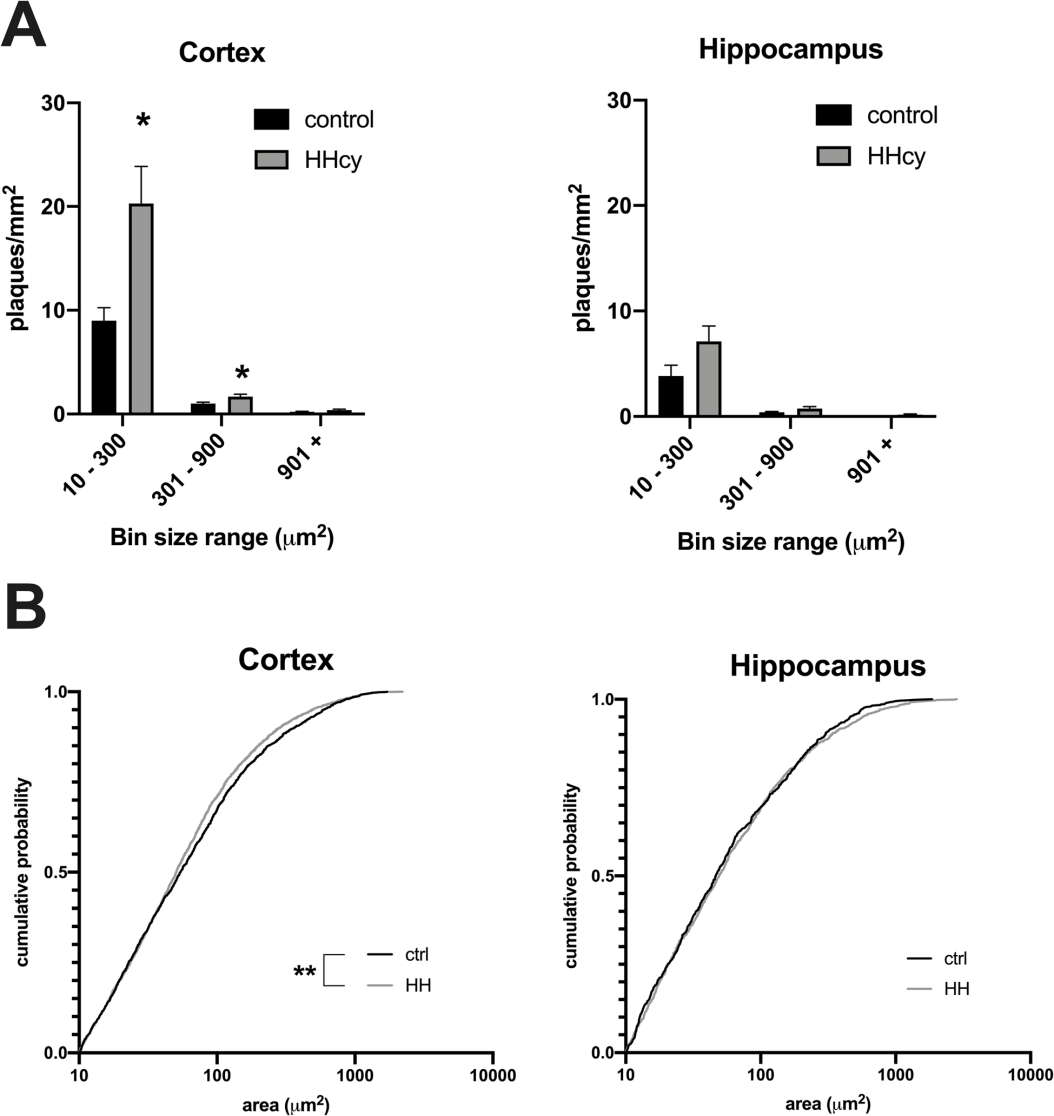
Dietary-induced HHcy alters the amyloid plaque size distribution in KI mice. **a** Plaques within each ROI were binned into three ranges: 10–300 μm^2^, 301–900 μm^2^, and 901 μm^2^ or greater, and compared across dietary condition. The average number of plaques in the first two ranges was significantly increased in the cortex of KI mice on HHcy versus control diet. Two-way mixed measures ANOVA with Sidak’s post hoc testing, *n* = 11 control and 10 HHcy mice, *p < .05. There was also an increase in small plaques in the hippocampus but the increase did not reach significance. **b** Cumulative probability histograms were generated for the complete set of analyzed plaques across animals within each dietary condition, showing that in the cortex there is a slight leftward shift in the distribution, indicative of an increase in smaller-sized plaques. Kolmogorov-Smirnov test, *n* = 1727 control and 3399 HHcy plaques in the cortex, and *n* = 741 control and 1264 plaques in the hippocampus, **p < .01

Next, amyloid plaque size analysis was performed in cortex and hippocampus in two ways. First, plaques were binned into small (10–300 μm^2^), medium (301–900 μm^2^), and large sizes (901+ μm^2^), with average plaques/mm^2^ in each bin compared across dietary conditions (Fig. 2a) [26]. This analysis showed an increase in smaller plaques in the cortex, consistent with increased plaque deposition (Fig. 2), as well as an increase in the medium-sized plaques possibly representing growth of extant plaques. The increase of plaque number in these bins did not reach statistical significance in the hippocampus. Next, cumulative probability distribution histograms were generated for plaque size in each dietary condition (Fig. 2b) [27]. In accordance with the binned data analysis, the distribution was significantly shifted leftward in the cortex of the HHcy versus the control KI group, indicative of a larger proportion of smaller plaques (Kolmogorov-Smirnov test, *p* = .0078). This was not significant in the hippocampus (Kolmogorov-Smirnov test, *p* = 0.78).

### Comorbid amyloid and HHcy pathologies subtly enhance pro-inflammatory cytokine levels

**Fig. 3:**
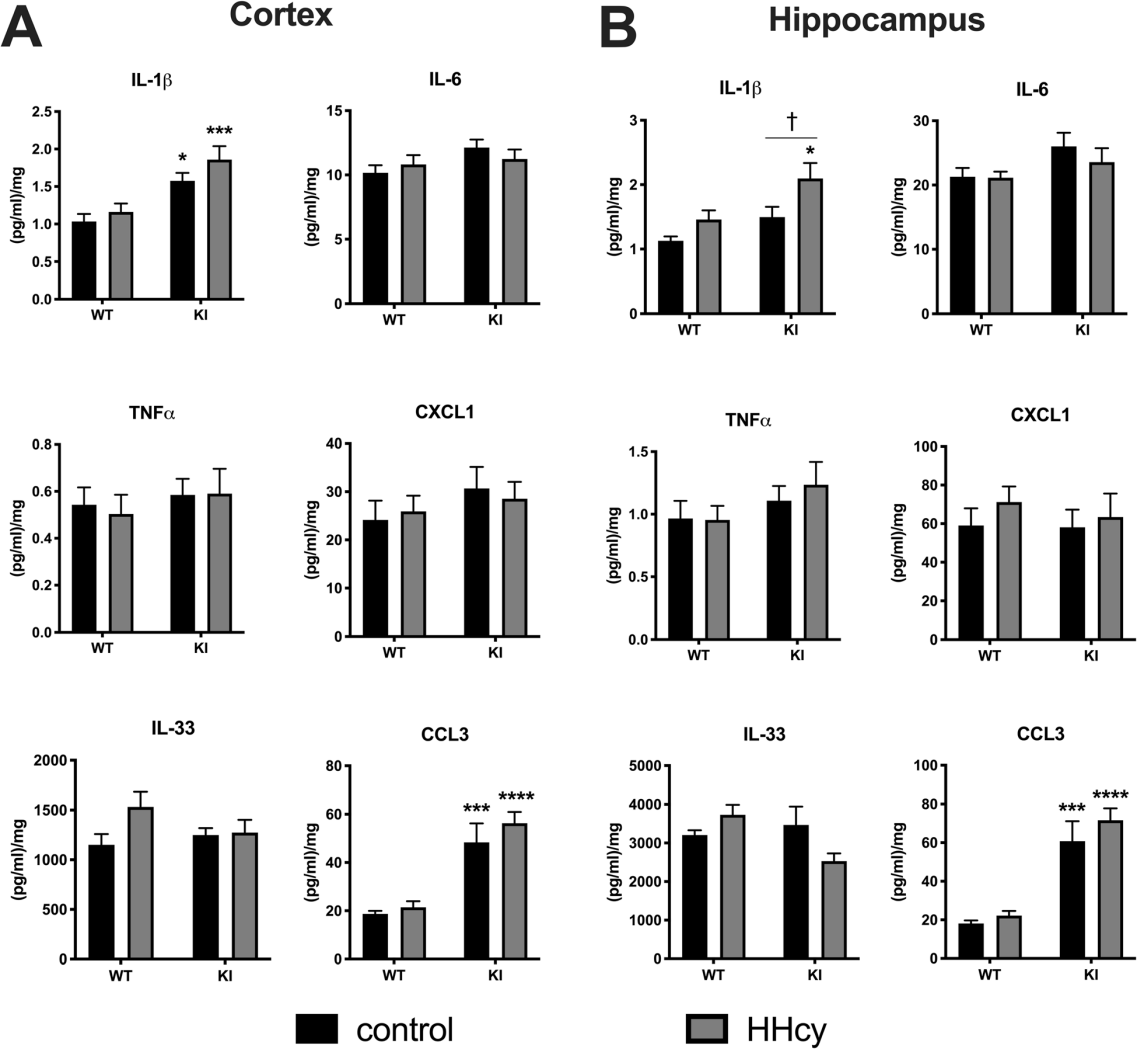
Dietary-induced HHcy increases hippocampal IL-1β in KI mice. Levels of cortical (**a**) and hippocampal (**b**) IL-1β, IL-6, TNFα, CXCL1, IL-33, and CCL3 are shown in response to diet and genotype. IL-1β levels in cortex are higher in KI mice compared to WT mice, but there is no additional effect of HHcy diet in this region. In contrast, IL-1β levels are significantly increased by HHcy diet in the hippocampus of KI mice but not WT mice. CCL3 was elevated in both cortex and hippocampus of KI versus WT mice, but diet did not alter the effect of genotype on this chemokine. *p < .05, ***p < .005, ****p < .001 versus corresponding WT dietary group, ^†^*p* < .05 versus KI control, two-way ANOVA with Sidak’s post hoc tests, *n* = 8–11 per group

To assess whether the increased amyloid plaque burden resulted in an exacerbation of pro-inflammatory responses, we measured levels of the inflammation-associated molecules CXCL1, CCL3, IL-33, TNFα, IL-6, and IL-1β by multiplexed MSD ELISA (Fig. 3). The overall changes in the cytokines measured were modest with respect to either strain or diet. There was a significant main effect of genotype on CCL3 expression in the cortex (*F*(1, 35) = 37, *p* < .0001) and hippocampus (*F*(1, 35) = 45.04, *p* < .0001), with significant differences detected by Sidak’s post hoc tests between KI and WT groups within both dietary conditions. This was also true of cortical IL-1β (*F*(1, 35) = 22.52, *p* < .0001). In the hippocampus, there was a significant main effect of genotype on IL-1β (*F*(1, 35) = 8.45, *p* = .0063) and also a significant main effect of diet (*F*(1, 35) = 7.26, *p* = .0108). Sidak’s post-hoc tests showed significant differences between the KI HHcy and WT HHcy groups as well as the KI HHcy and KI control groups (Fig. 3b). No significant differences were detected in levels of CXCL1, TNFα, or IL-6 in either region as a function of genotype or diet, but there was a significant diet by genotype interaction detected in the hippocampal IL-33 data (*F*(1, 35) = 5.08, *p* = .0306). Although the interaction was significant, post hoc tests for differences between KI and WT control or KI HHcy and KI control groups did not reach statistical significance.

### HHcy does not alter P2ry12 staining patterns in response to amyloid pathology

**Fig. 4:**
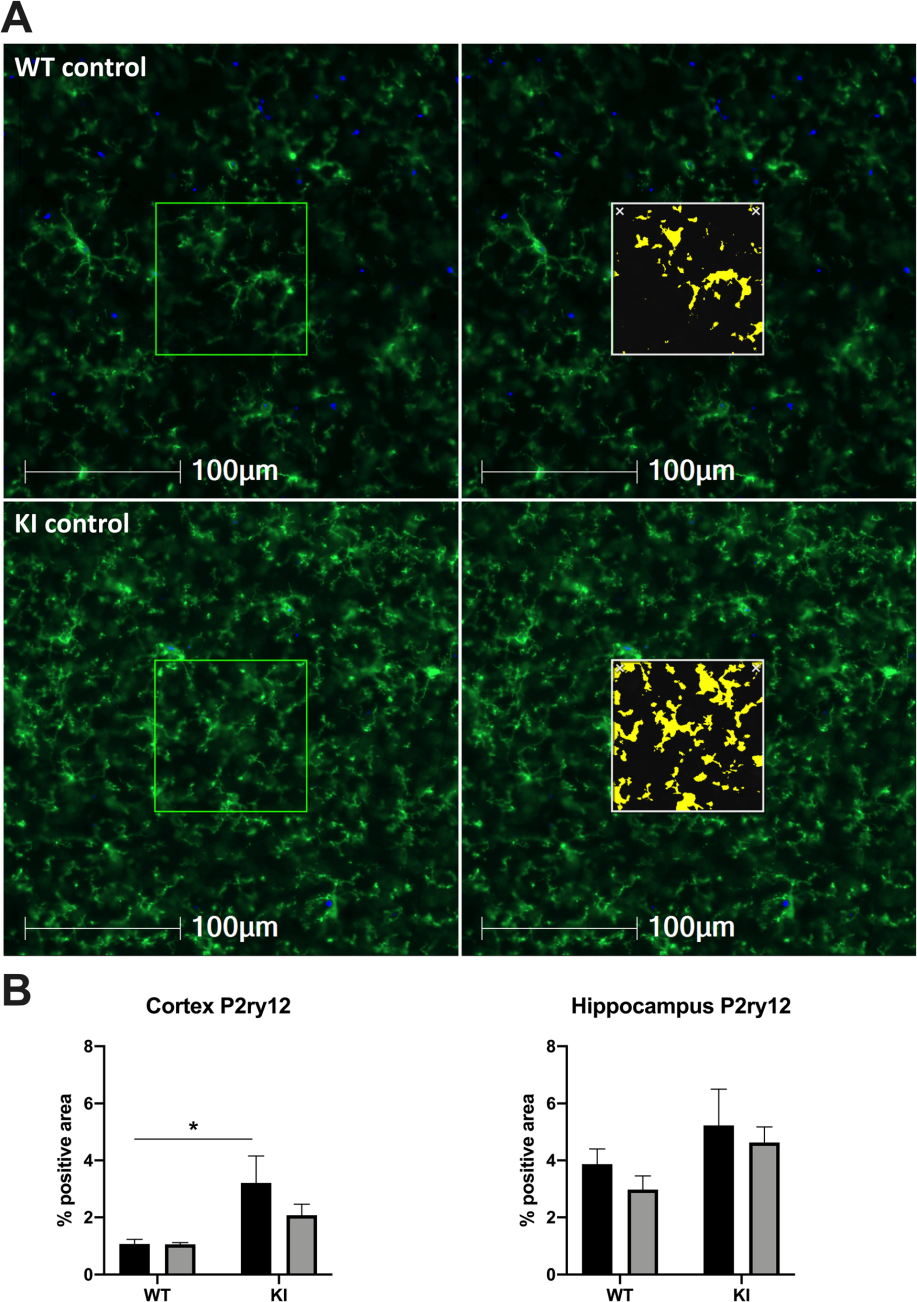
Dietary-induced HHcy is associated with a moderate decrease in cortical P2ry12 staining in KI mice. Representative images of cortex from a WT mouse on control diet (WT control) and KI mouse on control diet (KI control) are shown in **a**, with P2ry12 in green and DAPI in blue. A measurement mask is shown with output of the analysis algorithm shown in yellow. Total % positive area is quantified for the cortex and hippocampus in **b**. *p < .05 versus WT control mice, two-way ANOVA with Sidak’s post hoc tests, *n* = 7–10 per group due to exclusion of slides with out-of-focus areas within the ROIs

We quantified cortical and hippocampal P2ry12 staining as a microglia-specific marker [29] (Fig. 4a) indicative of overall microgliosis without potential inadvertent measurement of infiltrating myeloid cells. There was a significant main effect of genotype on P2ry12 staining in the cortex (*F*(1, 29) = 9.973, *p* = 0.0037), but no differences found in the hippocampus (Fig. 4b). A modest decrease in P2ry12 staining was observed in the cortex of KI mice on HHcy versus control diet; however, the difference between KI mice on HHcy diet and KI mice on control diet was not statistically significant (*p* = 0.08).

**Fig. 5:**
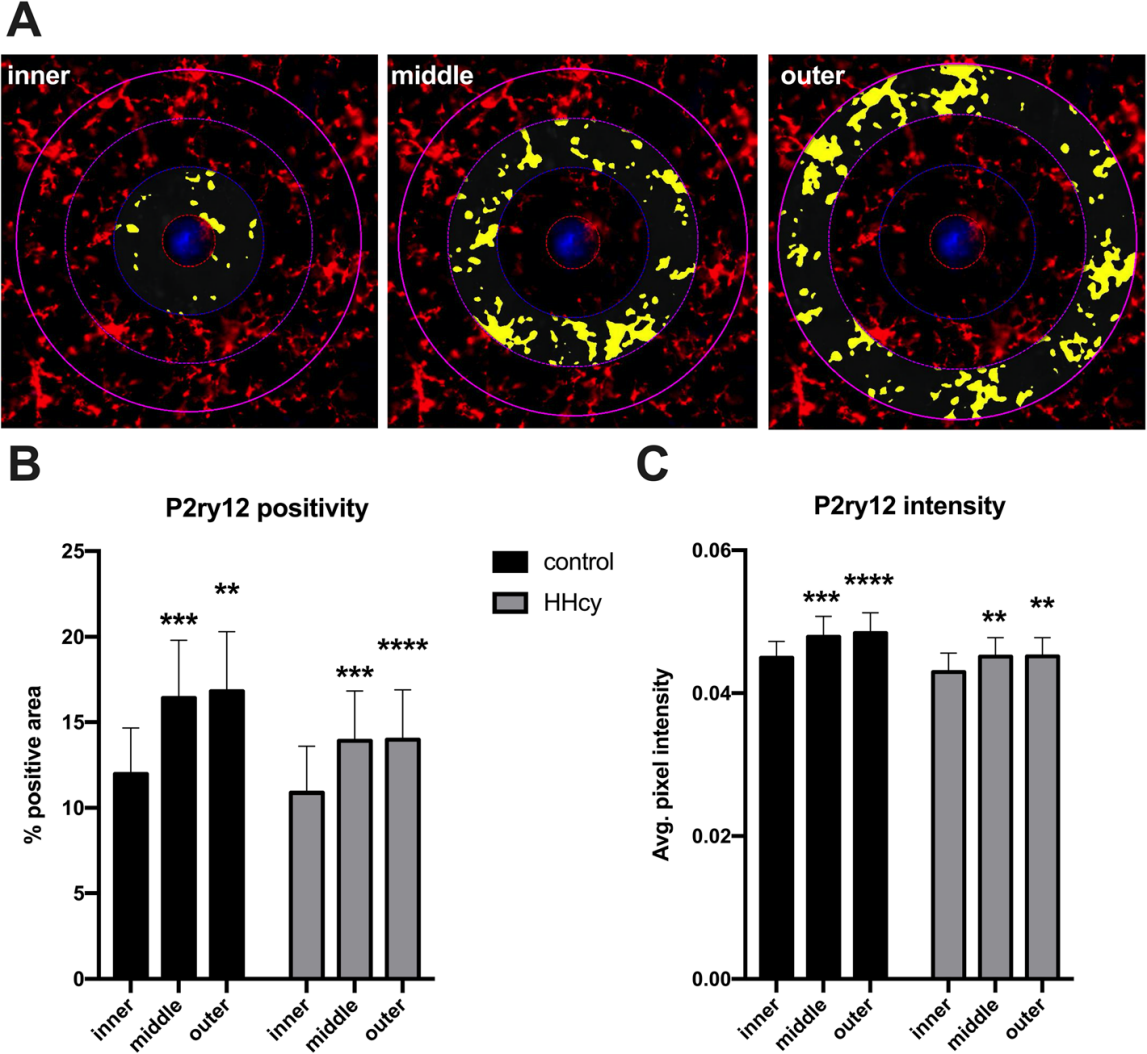
Spatial analysis of P2ry12 positive microglia near plaques in KI. Representative images of concentric, 30-μm width analysis regions are shown in **a** where P2ry12 staining is in red, 6E10 staining is in blue, and output of the analysis algorithm is shown in yellow. Total % positive area for P2ry12 is quantified in **b**, and average pixel intensity (AU) within each region shown in **c**. **p < .01, ***p < .005, ****p < .001 versus the inner ring within dietary groups. Two-way mixed measures ANOVA with Dunnett’s post hoc tests

Given the established downregulation of P2ry12 in plaque-associated microglia [15, 30], the subtle decrease in P2ry12 positivity in the cortex might be attributable either to a straightforward increase in plaque burden, to a further downregulation of P2ry12 in plaque-associated microglia when HHcy is present, or both. To clarify these possibilities, we performed spatial analyses of P2ry12 and 6E10 plaque staining. We found that the percent area double-positive for P2ry12 and Aβ 6E10 staining was unaltered within cortex between KI control (*M* = .018%, SD = .015) and KI HHcy mice (*M* = .018%, .012) (*t*(18) = 0.0897, *p* = 0.9295). This was similarly true in the hippocampus of the KI control (*M* = .013%, SD = .009) and HHcy mice (*M* = .014%, SD = .011) (*t*(18) = 0.1474, *p* = 0.8843). To examine the relationship more closely, a spatial proximity analysis was performed around a subset of plaques in the cortex (Fig. 5a). Results were consistent with a lack of dietary effect on the spatial distribution of P2ry12 staining in response to amyloid plaques. This was true when assessed either by total % positive area of P2ry12 staining (Fig. 5b) or average pixel intensity (Fig. 5c). The relative dearth of P2ry12 staining and intensity in the inner ring closest to the plaque is consistent with previous reports of P2ry12 downregulation near plaques [15, 30]. Taken together, these data indicate that the small decline in overall cortical P2ry12 staining may be primarily attributable to an increase in overall plaque number.

### HHcy modifies microglia-associated gene expression in WT and KI mice

**Fig. 6:**
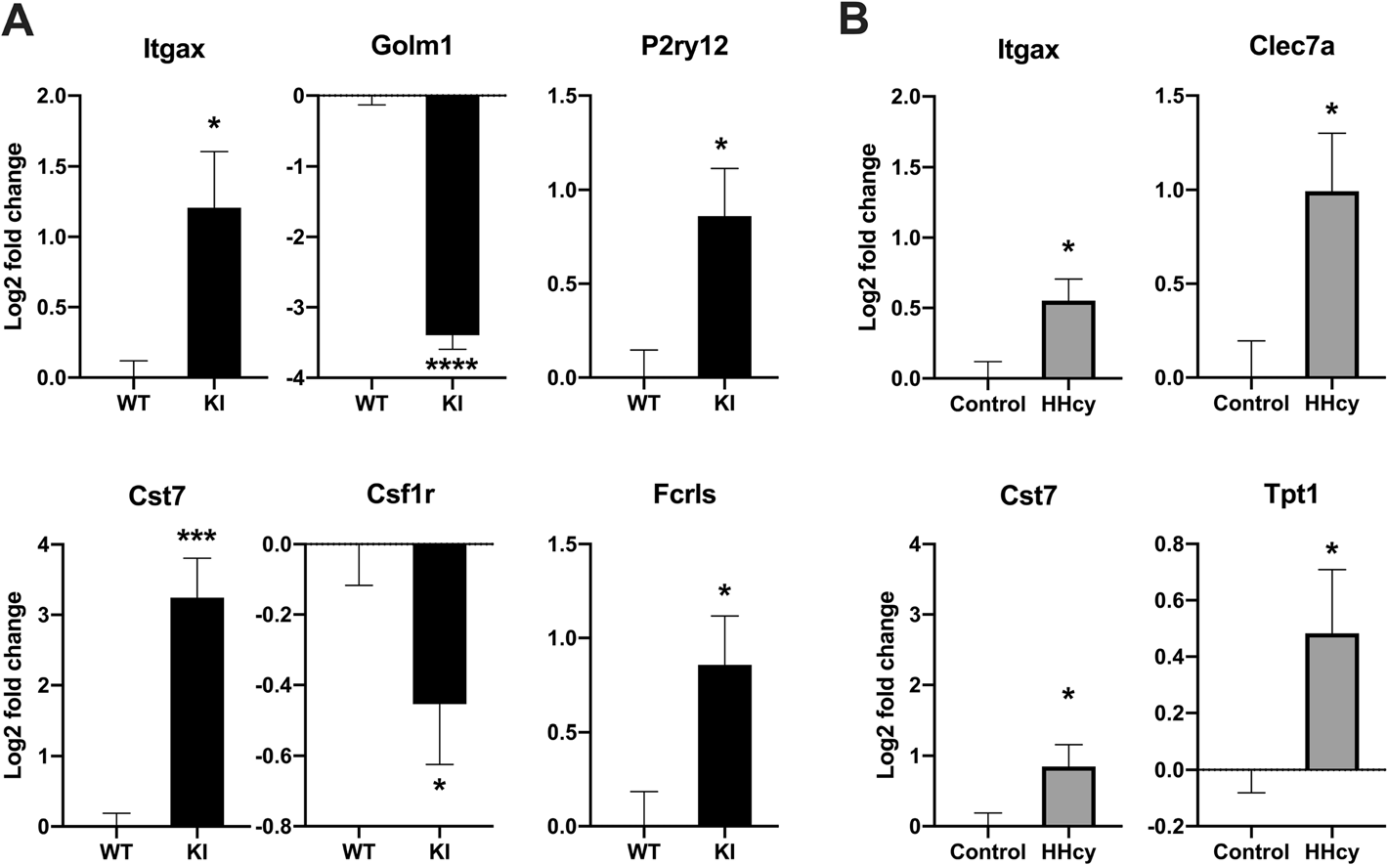
Gene transcript levels are significantly altered in the single pathology amyloid or HHcy contexts. Gene transcripts significantly altered between KI mice on control diet and WT mice on control diet are shown in **a**, and transcripts significantly altered between WT mice on HHcy diet and WT mice on control diet are shown in **b**. Transcript fold changes are log2 normalized in comparison to the WT control diet condition. *p < .05, ***p < .005, ****p < .0001, multiple *t* tests, uncorrected

To begin exploring possible additive or interactive gene expression changes that occur in the comorbidity context, we measured whole-tissue cortical changes across a panel of 50 genes implicated in microglial responses to amyloid pathology (Table 1). A subset of 8 randomly selected mice per group (32 total samples) were used for the gene expression analysis, with data normalized to the expression level of WT mice. Chil3 is excluded from statistical analysis because two of the samples did not show any detectable level of this gene (see supplement 3 for full dataset). We first assessed whether our panel, despite not being run on RNA extracted from purified microglial populations, could detect any of the expected increase in DAM markers and decrease in homeostatic markers in the KI control vs WT control comparison. We found that 6 genes were detectably altered (Fig. 6a). In line with expectations, the DAM markers Itgax and Cst7 were upregulated and the homeostatic markers Golm1 and Csf1r were downregulated. Interestingly, the homeostatic markers Fcrls and P2ry12 were found to be upregulated, which could be indicative of an overall increase in the number of microglia.

**Fig. 7:**
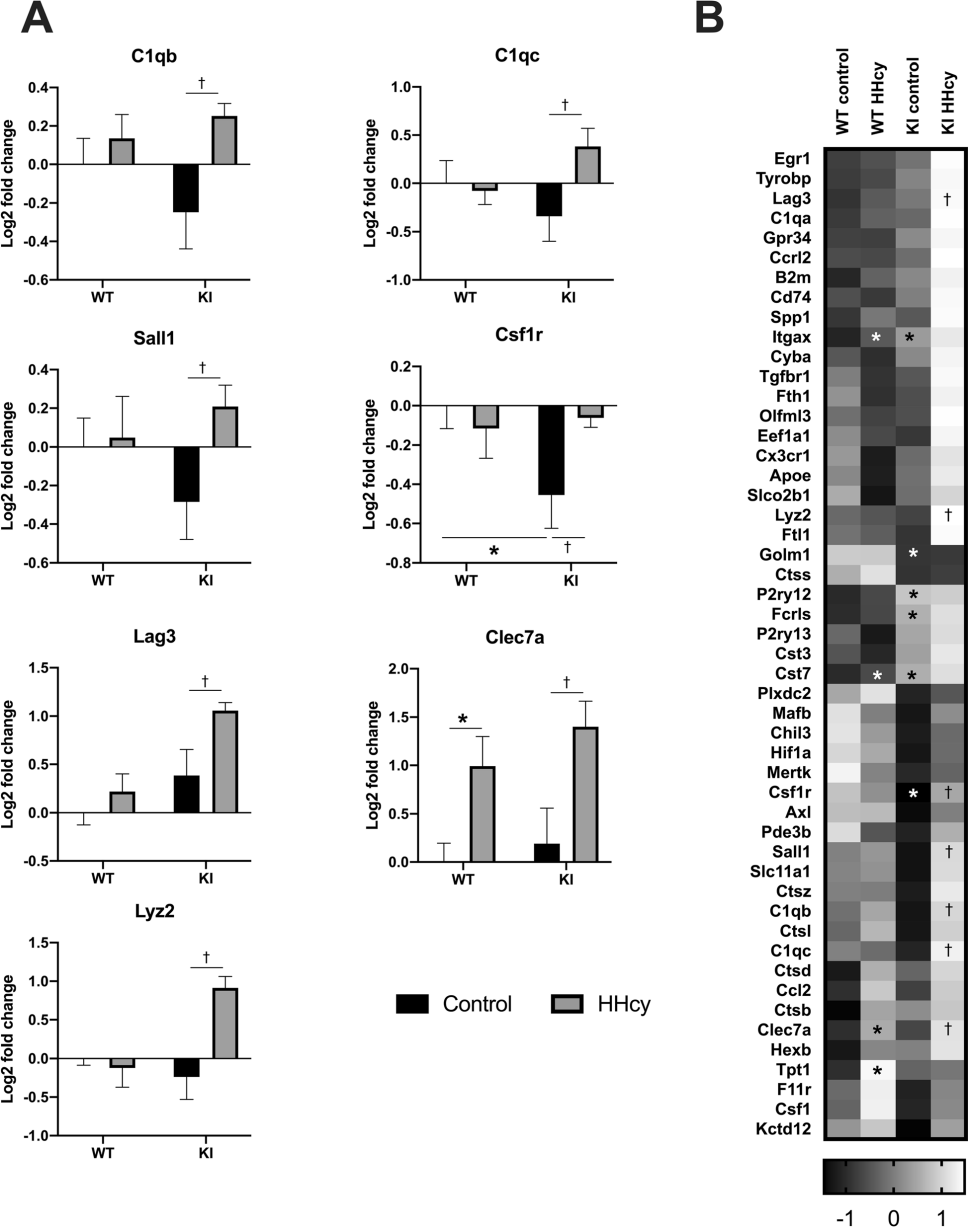
Gene transcript levels are significantly altered in the amyloid and HHcy comorbidity condition. Graphs displaying log2 fold change values of all four groups for the 7 genes differentially expressed between KI control and KI HHcy mice are shown in **a**. A heatmap summarizing *z*-score transformed means of each gene across the 4 groups is displayed in **b**. *p < .05 versus WT control, ^†^*p* < .05 versus KI control, multiple *t* tests, uncorrected

When comparing WT HHcy and WT control groups, we found 4 significantly upregulated DAM markers: Itgax, Cst7, Clec7a, and Tpt1 (Fig. 6b). To determine whether there was an overall increase in the DAM signal or evidence of unique transcriptional changes, we next compared the KI HHcy and KI control groups. Seven genes were significant within this comparison, and the average transcript levels of these genes across all 4 groups are displayed in Fig. 7a. Interestingly, not only some damage-associated genes (Lag3, Clec7a, Lyz2) but also putative homeostatic markers (C1qb, C1qc, Sall1, Csf1r) were upregulated in the comorbid condition relative to the KI control group. A summary heatmap of *z*-scores with significant comparisons is shown in Fig. 7b. The full gene expression dataset is provided in supplement 3, and graphical representations of the processed data in supplement 4. These findings indicate that the comorbid pathologies have complex interactive effects in addition to straightforwardly additive ones. Replication and extension of these findings using unbiased whole-transcriptome analyses in isolated microglial (and other) cells will be an important next step in this line of research.

## Discussion

We report here several major findings that increase our understanding of physiological changes occurring in the relatively common context of comorbid amyloid- and HHcy-associated pathologies, and also lay the groundwork for follow-up studies. First, 8 weeks of HHcy-inducing diet is sufficient to produce a large increase in parenchymal plaque deposition, particularly smaller plaques, without a concomitant change in levels of soluble Aβ. This implies that interstitial levels of soluble Aβ are being kept in equilibrium via increased plaque formation, consistent with the notion that sequestration into plaques can be a protective response against the more toxic soluble Aβ species [31]. Our data align with the hypothesis that HHcy can enhance AD risk by increasing overall amyloid burden, a finding that has been replicated across models of AD and under different methods of HHcy-induction [21, 32–34]. Importantly, this appears reflective of what occurs in patient populations, where plasma Aβ positively correlates with homocysteine levels and elevated Hcy is also associated with higher brain Aβ accumulation and CAA pathology [35–38].

Second, HHcy in this model has a small effect on the pro-inflammatory milieu, at least in terms of the factors measured in this study. Partially this may be due to our focus on a limited subset of cytokines chosen primarily for reported responsiveness to amyloid pathology. Within this subset, there was only a modest pro-inflammatory cytokine response overall with CCL3 and IL-1β elevations driven by genotype. Only IL-1β was increased due to HHcy diet and even this effect was observed only within the hippocampus. Given the lack of a similar effect in the cortex, it appears that HHcy does not alter IL-1β levels primarily via increased amyloid. That the hippocampus is uniquely susceptible to exacerbated inflammatory responses in the comorbid context is intriguing and may be related to its sensitivity to vascular insults, particularly disruptions to the blood-brain barrier [39], which is a well-described feature of this particular model of HHcy [21, 25]. Interestingly, there was also a significant diet by genotype effect on hippocampal levels of IL-33, although the post hoc comparisons were non-significant potentially indicating a lack of adequate statistical power for this comparison. Biologically relevant changes in this pathway may be worth exploring in future experiments.

Overall, the cytokine data are consistent with previous studies that indicate an enhancement of pro-inflammatory responses in either WT or AD model mice in response to HHcy [20–22, 32]. Despite this, the magnitude of the effect appears to be much smaller in the present case, and there are several possibilities for this apparent discrepancy. Notably, the increase in plasma Hcy level was comparatively less severe in the present study. This might indicate that the pro-inflammatory response is graded depending upon the severity of induced HHcy. The implication of this scenario is that patients with severe HHcy may require treatments suppressing neuroinflammation; those with moderate HHcy may benefit more from alternative approaches. Alternatively, the discrepancy might simply be due to the method of cytokine measurement employed. Previous studies relied on transcriptional changes as indicative of cytokine modulation, but the present study more directly measured protein levels. Because cytokines are subject to complex regulation at many levels, large changes in cytokine mRNA transcripts may or may not represent changes at the functional protein level. For example, in a previous study that measured cortical IL-1β in this KI mouse strain, a 7-fold increase in IL-1β mRNA occurred alongside a much more modest 20% increase in level of the mature protein [26]. Over-reliance on cytokine transcript levels without confirmation of protein changes may therefore overestimate or otherwise misrepresent the actual pro-inflammatory changes in a given brain region or pathological context. Such possibilities warrant more complete characterization of changes to the neuroinflammatory milieu occurring at different levels of Hcy elevation, including the relevant transcriptional or translational regulatory mechanisms across contexts.

Although the neuroinflammatory response was relatively mild in this model, there were several important findings with regard to microglia. When P2ry12 staining was taken as a marker of microgliosis, we observed the expected increase in staining within the cortex of KI versus WT mice, alongside a parallel increase in P2ry12 mRNA level. Interestingly, there was a moderate decrease in P2ry12 staining in the KI mice on HHcy diet relative to those on control diet. No such effect was observed in the WT animals, indicating that the reduction of P2ry12 staining in the comorbidity model might be primarily responding to the increase in plaque burden. Consistent with this, the expected downregulation of P2ry12 staining as a function of proximity to plaques was observed [15, 30] and was unchanged by diet.

To further interrogate the relationship between these pathologies and microglial phenotype, a subset of microglia-enriched genes was evaluated. Fifty genes were selected, chosen based upon recently published datasets derived from microglia isolated from amyloid-overexpressing mice [15, 16]. Half of the selected markers are positively regulated due to amyloid pathology and considered to be DAM markers. The other half are negatively regulated by amyloid pathology and considered to be homeostatic markers. We sampled both under the expectation of detecting an increase in some of the damage markers and a decrease in some of the homeostatic markers, which is precisely what we observed in comparisons between the KI control and WT control mice. The damage-associated genes Itgax and Cst7 were upregulated, and the homeostatic genes Golm1 and Csf1r were downregulated. Unexpectedly, the homeostatic genes P2ry12 and Fcrls were found to be elevated rather than reduced in the KI mice. This may be reflective of enhanced microgliosis in response to amyloid overshadowing more subtle effects occurring in the relatively small subset of plaque-associated microglia. That more genes were not detected as being significantly altered in our panel is likely due to some combination of a lack of a cell-specific signature, the attenuated amyloidosis that occurs in this model relative to the overexpression models more frequently utilized (e.g., 5xFAD mice), heterogeneity resulting from the use of a different background strain [40], or the unbalanced inclusion of both sexes and potentially increased biological variability of the sample. Although sex differences have not been studied in the KI mice, their existence is plausible given that sexual dimorphism has been observed in some other strains carrying APPswe/PS1dE9 mutations [41]. Further, we have found some indications that female mice might be differentially susceptible to the deleterious effects of this particular HHcy diet [25]. Given the sexual dimorphism that exists clinically in both AD and HHcy contexts, it will be important to isolate sex as a variable in future studies.

In addition to the transcripts responsive to amyloid, we found 4 responsive to HHcy diet in the WT mice: Itgax, Cst7, Clec7a, and Tpt1. That Itgax and Cst7 are upregulated due to HHcy as well as amyloid is consistent with the frequently described elevation of these genes across pathological contexts [42]. Clec7a is notable in that little response of this transcript was detectable in the KI control mice relative to WT control. Clec7a is a pathogen-associated molecular pattern receptor involved in the innate immune response, including microglial phagocytic responses [43], and it has been shown to increase in parallel with plaque burden in mouse models of AD [44]. The most parsimonious explanation for these data is that HHcy increases Clec7a mRNA in a wider population of microglia throughout the cortex, whereas plaque deposition is associated with increased Clec7a mRNA primarily in those microglia that are closely plaque-associated. When amyloid and HHcy co-occur, there may be both an increase in Clec7a mRNA across the wider microglial population, as well as an increase in the proportion of microglia that are plaque-associated (due to the enhanced amyloid burden). Interestingly, Clec7a has been reported to respond to neuronal damage rather than the presence of Aβ per se [15]. The HHcy diet may therefore be causing significant neuronal damage, and a closer examination of the effect of the HHcy diet on neuronal health and functioning in relationship to microglial parameters in both the WT and KI conditions should be investigated in future work. Tpt1 is a gene implicated in the cellular DNA damage response, known to be upregulated in the context of folic acid deficiency and homocysteine elevation (and amyloid itself) [45]. That this response was not observed in the KI mice with HHcy might be due to the pre-existing pathway activation due to the presence of amyloid. Taken together, these data indicate that the gene expression panel is successfully detecting expected pathology-associated changes in gene expression levels when applied to the whole tissue.

Regarding the primary question of how microglia react in the comorbidity context, there are several possibilities that would predict different patterns in the data. For example, if the microglia were primarily responding to a straightforward increase in amyloid burden, then the prediction would be further upregulation or downregulation of the amyloid-associated transcriptional changes identified in the KI vs WT comparison. Evidence for this was not detected in the present study, and this could imply that certain microglial responses are already maximally engaged by one pathology or the other. Alternatively, overall plaque number may not be the primary driver of these shifts but, instead, levels of soluble Aβ may be more important. There is also an indication that more of the microglia are in a DAM state when both pathologies are present, as some DAM transcripts not significantly elevated in either the HHcy or amyloid conditions alone were detectably elevated only in the comorbid condition: Lag3 and Lyz2. Further, many other genes displayed that pattern even though statistical significance was not reached. Interestingly, several homeostatic genes showed a divergent directionality between the single factor pathologies and the comorbid condition, indicative of potentially interesting responses unique to the comorbid context: C1qb, C1qc, Sall1, and Csf1r.

C1qb and C1qc encode parts of complement component 1q (C1q), which plays a critical role in synaptic pruning during development and in pathological conditions such as AD [46]. Although C1qa transcript levels were not significantly altered in any comparisons, the overall pattern suggests that there may be enhanced synaptic loss or neuronal dysfunction in the comorbid condition, a possibility worthy of exploration. Sall1 is a transcription factor that under physiological conditions inhibits microglial activation and pro-inflammatory responses [47]. Its upregulation in this context suggests reduced microglial reactivity to amyloid, and this may help to explain the modest effects on the pro-inflammatory cytokines measured. Csf1r signaling is well-characterized for its involvement in microglial proliferation and survival [48]. Recent studies have shown that chronic pharmacologic suppression of this receptor results in loss of microglia concomitant with protection against neurodegenerative changes in the 5xFAD amyloidosis model [49, 50], and its relative increase might be detrimental in this context. Although the specific implication of transcriptional changes in any particular gene is not clear, when taken together, these data indicate nonetheless that there exists both a potentiation of microglial-associated stress responses as well as more complex emergent responses in the comorbid Aβ and HHcy condition. Future studies utilizing more animals of both sexes, specific cellular isolation techniques, and unbiased measurement of whole-transcriptome changes will be useful in clarifying the microglia-specific alterations in this mixed pathology model. Ultimately, data must also be compared to samples from relevant patient populations to assess the external validity of the findings.

## Conclusion

Modulation of microglial function represents a promising area for therapeutic development in Alzheimer’s disease research. Given the frequent co-occurrence of multiple pathologies in patients with dementia, the success of this approach will likely require a deeper understanding of microglial responses in various contexts. The present study supports such efforts by offering an initial characterization of both general and unique microglial-associated changes in response to comorbid amyloidosis and HHcy. Whether successful treatments will focus on ameliorating general microglial dysfunction, will be tailored to specific responses, or both is an open question. The present study contributes to the groundwork necessary for future studies to explore this topic in more depth.

## Supplementary information

**Additional file 1:.** Supplemental figure 1: Tissue dissection and regions of interest for analyses.

**Additional file 2:.** Supplemental figure 2: HHcy model validation data.

**Additional file 3:.** Full gene expression raw dataset.

**Additional file 4:.** Full gene expression graphical dataset.

## Data Availability

The datasets generated and analyzed for this study are available from the corresponding author upon reasonable request.

## Abbreviations

Aβ: Amyloid beta
AD: Alzheimer’s disease
ANOVA: Analysis of variance
C1q: Complement component 1q
CAA: Cerebral amyloid angiopathy
Hcy: Homocysteine
HHcy: Hyperhomocysteinemia
KI: Knock-in
MSD: MesoScale Discovery
PBS: Phosphate-buffered saline
qRT-PCR: Quantitative reverse-transcriptase polymerase chain reaction
SD: Standard deviation
WT: Wildtype
