# Supplementary material for: Microglial-associated responses to comorbid amyloid pathology and hyperhomocysteinemia in an aged knock-in mouse model of Alzheimer’s disease
Source: J Neuroinflammation. 2020 Sep 17;17:274. doi: 10.1186/s12974-020-01938-7 (PMC7499995; doi:10.1186/s12974-020-01938-7)
Supplement: Supplementary file 1 — Additional file 1:. Supplemental figure 1: Tissue dissection and regions of interest for analyses. [file 12974_2020_1938_MOESM1_ESM.docx]

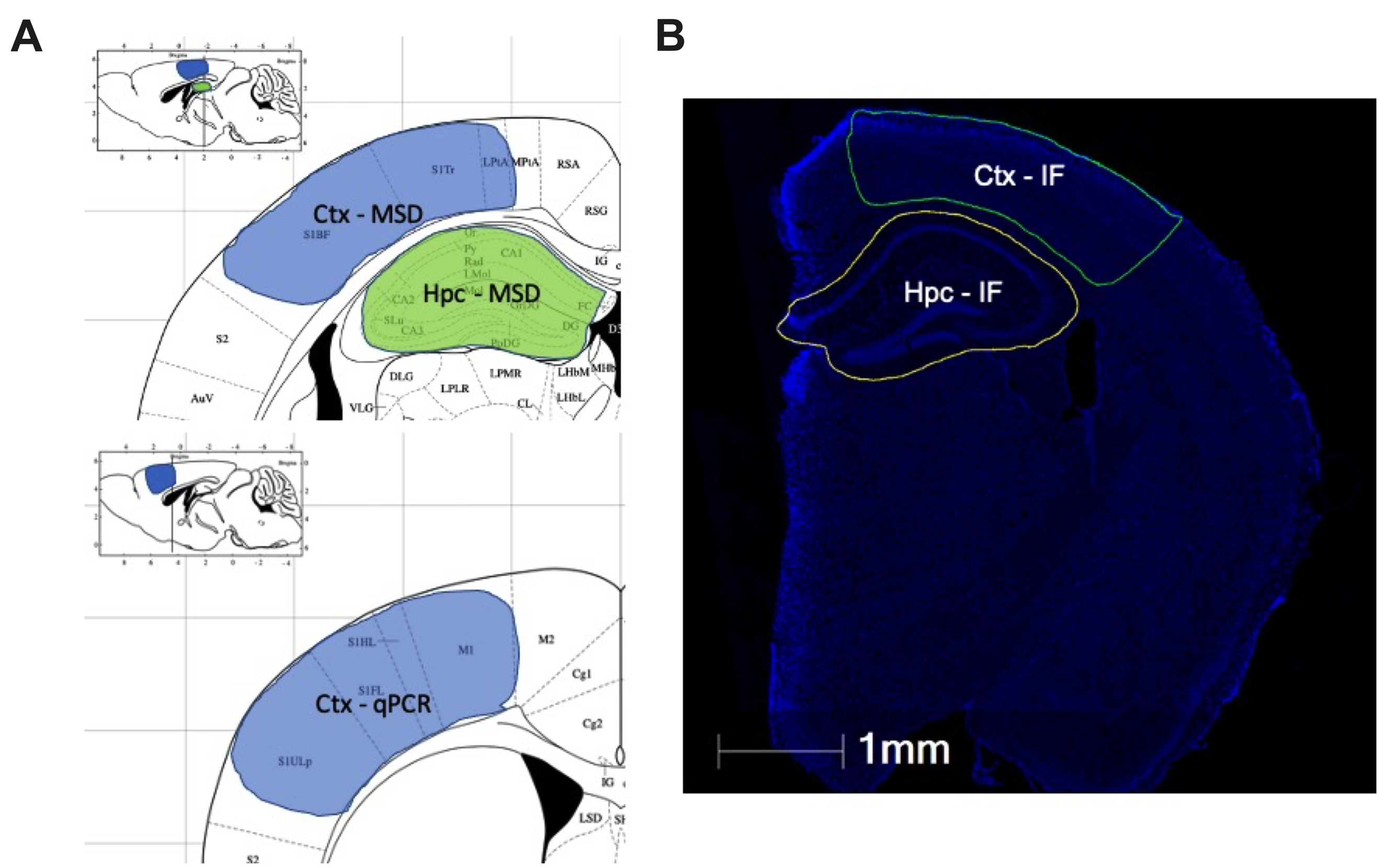


**Supplemental figure 1: Regions of interest for biochemical and immunofluorescence analyses.** (A) Representation of dissected cortical and hippocampal tissue pieces from the left hemisphere used for MSD (above) or qPCR analysis (below). For MSD, a portion of the dorsal hippocampus or the overlying cortex was homogenized. For qPCR, an area of cortex just rostral to that used for MSD was taken. (B) Sample image of outlined hippocampal and cortical regions used in the IF analyses. Image shows DAPI staining in blue with scale bar for reference.
