# Supplementary material for: Microglial-associated responses to comorbid amyloid pathology and hyperhomocysteinemia in an aged knock-in mouse model of Alzheimer’s disease
Source: J Neuroinflammation. 2020 Sep 17;17:274. doi: 10.1186/s12974-020-01938-7 (PMC7499995; doi:10.1186/s12974-020-01938-7)
Supplement: Supplementary file 2 — Additional file 2:. Supplemental figure 2: HHcy model validation data. [file 12974_2020_1938_MOESM2_ESM.docx]

**Supplemental figure 2: HHcy validation data.** (A) Weight loss associated with 8 weeks of HHcy diet is shown, alongside weight change curves. ****p < .0001 vs within-genotype control diet comparison. Mice on HHcy diet lost significant weight regardless of genotype, but this effect was larger in the WT mice, *p = 0.0118. (B) Baseline differences in weight are shown between males and females of each genotype. There was a significant main effect of genotype (F(1, 35) = 28, p < .0001) and a sex by genotype interaction (F(1, 35) = 4.6, p = 0.037). There were no within-genotype differences by sex. (C) Homocysteine levels are not significantly different between WT and KI mice on HHcy diet, student’s t-test, n = 5 per group. (D) A subset of WT mice on control and HHcy had plasma measurements taken of hemoglobin and hematocrit, with significant declines in both after 8 weeks on HHcy diet. **p < .005, student’s t-test, n = 3 control and 4 HHcy.
